# Address

**Published:** 1877-05

**Authors:** R. G. Thomas


### ADDRESS.

*Before the Senior Class of the Dental College of the University of Michigan. March* 27, 1877.

By Dr. R. G. Thomas.

Among the many things I desire to impress indelibly upon your minds, *this* is not the least. *Do not be in haste to make money.* For if this be your highest ambition, all your powers for scientific research will be laid, *not upon*, but under the altar. The errors in your practice will greatly multiply before your vision as you advance in years, but let not *this* be one of them, establish first your reputation, and let money be a secondary consideration.

Let your ambition be to *make patients*,—and you may spell it both with a t and a c,—and in using the word *make*, I refer to quality rather than to quantity. If you succeed in bringing about you, patients who are intelligent and discriminating, and they will be, just in proportion as you are able and inclined to make them such—the state of your finances may be among the least of your troubles.

When you have decided upon a place to locate, my advice is not to place your office in the most public building you can find, especially if in a large city, for the persons who call upon you for your services because they happen to see the sign of *dentist*, are not likely to be of much real value, they will not be discriminating to say the least, although you will occasionally meet people in this way, of whom you will be able to make valuable patients.

Locate your office in a place a little remote, and retired from the publicity of business; a place that is in an atmosphere of quiet and reserve, rather than of public preferment. My preference is decidedly in favor of a private dwelling for an office. It posesses many advantages over a public building; one of which is freedom from noise occasioned by rambling through long and uncarpeted halls; and you will doubtless find that quiet is very essential to the comfortable practice of dentistry.

Another advantage is comparative freedom from those *friendly friends* who just drop in to see you a *minute*, and stay an *hour*; with no other aim than to pass away time; and just here I would call your special attention to the importance of the proper disposition of your time. Do not loaf yourself, neither allow others to loaf about you. In the beginning of your practice, some of you will doubtless find time hang heavily upon your hands. To such I would say, lay out for yourselves some special line of work, physical or mental, theoretical or practical; but let it be something that not only will engage your mind, and keep you advancing with the profession, but will give your friends to understand that you are engaged, that you are not idle; paucity of patients is no excuse for idleness in one who is engaged in a profession so full of revealed and unrevealed mysteries as ours. I believe nothing will cause a community to place a low estimate upon a young professional man's capabilities sooner, than to see him idling away his time, or any portion of it, in this place or that, with no special object. Systemize your office hours; do not allow people to come to your office and habitually find that you are *out*, when you should be *in*, neither allow yourself always to be *in*, thereby giving people to understand that they can find you at any and all times. You will need daily out-door exercise, and let it be regularly and faithfully attended to.

I have made a great saving of time and annoyance, both to myself and patients, by setting apart an hour each day for consultation. I have chosen from 2 to 3 o'clock, during which time I do not try to operate. No man can with propriety, or justice leave his patient in the midst of an operation to diagnose a case. It is not just to the patient you are operating for, to leave the chair for any length of time; your time is theirs, they are to pay for it. Neither can you do justice to the case you are called upon to diagnose; for you are too well aware that you are taking your patient's time, consequently your diagnosis will be rapid and incomplete.

In answer to this you may say, "I am beginning now, and shall only be too glad to see patients at any time that is most convenient to them." True, but they can, and will make it convenient to come at your hour, if they know you have one. Publish on your appointment card that your consulting hour will be from such to such a time each day, if you begin in this way, it will be much better than to wait till some future time. I have to regret now that I was not wise enough to begin it when I commenced practice, for a trial of the past year and a half has enabled me to realize in a measure, how much valuable time has been lost both to myself and patients, by seeing people during operating hours.

In selecting your rooms look more after convenience and health-giving facilities, than for grandeur and display. Let your surroundings be commensurable with your means, for it will be far more agreeable to take a step in advance than *vice versa*; and your patients will commend it as well.

Avoid doing violence to your reputation by getting into debt. If you cannot afford but one room, be content with that till you can afford more, but let that *one* be well chosen, and well kept after it *is* chosen. Do not accept the prevailing idea that your room must be located in the northwest corner of the building, thereby depriving yourself of that choicest of blessings, *sunlight.* I operated for eight years with my chair facing directly south. The sun's rays were controlled by white muslin curtains hung from both top and bottom of the window; and many a day have I revived my drooping spirits and wasted nerve forces by taking a sun bath, when opportunity afforded, although at that time "blue glass" was a thing unheard of. Doubtless all of you have observed how much more cheerful is a room with a southern outlook, than one facing in any other direction. Walk along the streets of any of our cities and you will observe that the large majority of vacant lots and unoccupied houses are located on the south side of the street looking north. At the approach of Spring you will also observe that the verdure on the north side of the street is at least ten days in advance of that on the other side. Therefore I say locate your rooms, if possible, with a south and east exposure.

As your acquaintance becomes extended with those practicing dentistry, you will notice how large a proportion are complaining of nervous prostration, and general indisposition. It being true that the practice is such as to closely confine us, it becomes all the more necessary that we should lay hold of all the hygienic agents we can command.

If your means will permit, you should have at least three rooms, (and more can be used to advantage) but if you can afford but *one* room, have it large enough to make into three apartments, dividing with low partitions. First of all locate your operating apartment, or the room you expect to spend most of your time in. Arrange for good ventilation. as well as proper heating facilities. If well ventilated, it need not be large, for a large operating room, is too much of an inducement for those waiting upon friends to seat themselves in, very much to your annoyance if there is more than one aside from the one you are operating for. Do not have more than one chair aside from your operating chair, as there should be no conversation in this room except that which is controlled by yourself.

Your next best apartment should be your laboratory. In this you should have a second operating chair,—if you cannot have a separate room purposely for it—where in case of absolute necessity you can slip a suffering one in (although you should not make a practice of it, *except* to relieve suffering) to extract a tooth or apply a remedy, and your patient be wholly unaware of your movements. *Never* ask your patient to relinquish the chair to accommodate another; some patients will do it without any manifest displeasure, but most of them will feel it, if they do not evince it. If you cannot afford a second operating chair improvise one, by putting a head-rest on a common chair.

Some one may object to a chair in the laboratory for the reason that it is not always in condition to take a person into. This would be one of the reasons why I should advise it; for if any of you should be so unfortunate as to need an incentive to keep your laboratory in good condition, this will probably be as good a medium as you can command. The larger this room the better.

My object in this lecture is not to advise you as to what instruments, and appliances you should put into these rooms, for you all know that now; if you do not, nothing that I can say will help you out of the difficulty. I rather desire to direct your attention to what may seem to you, secondary matters, but nevertheless, matters that go to make complete the practice of the profession you have chosen.

Your third, and least important, will be your waiting room. This, like the others, should be kept thoroughly *clean*. What you have in it should be an indication of your good taste, rather than the extent of your purse; but as your purse expands, let it manifest itself in the quality of your instruments, and appliances rather than in fine furniture and ornaments.

In your operating room, your instruments should be so arranged as to enable you to facilitate business as much as possible:—not that I would advocate rapid operating, for the sake of rapidity,—but let your instruments be so arranged that you will spend no time needlessly looking for, and handling them. You should make yourself so familiar with the locality of each instrument, that you can pick it up in the dark if need be. And when not in use they should be kept out of sight. I have known a few men in the profession, (and have heard of more) who seemed to have a peculiar desire for a display of instruments, apprising nervous women and timid children of the unwelcome operations awaiting them.

Keep all your instruments *scrupulously* clean, nothing will disgust a patient quicker, than to see instruments laid out before them, bearing upon their points the debris from another's mouth. Always *clean* your instruments and put them in place at the close of each patient's sitting, even if you expect to use them the next minute. By observing this rule you will avoid the mortification of being reminded, unexpectedly some times when inviting a patient to your chair that "these instruments have just been in some one else's mouth."

Always have a good supply of towels, napkins, soap and water at your command. Short and clean finger nails, a breath and brain free from *tobacco* and *whisky*, a conscience "void of offence," and a poise of judgment, ready, and reliable.

I would advise you to avoid purchasing instruments in sets, simply because dealers present them to you in that way. Buy only such as necessity demands. In this way you will secure a collection that will be of value, and also avoid cumbering your cases with those you never use. Keep your instruments sharp: and for this purpose a good Arkansas stone is the best, and should be your daily companion.

Just here I desire especially to impress upon you the importance of thoroughly drying your cavity before you commence the preparation of it, for your patients benefit as well as your own. To my mind it is very apparent that the sensitiveness present in the dentine, is not unlike the electric current; and as water is among the best conductors of electricity, so the saliva in a cavity, acts as a medium to convey the sensations to all parts exposed; although your cutting is confined to a very small portion: while if your cavity is kept thoroughly dry, the sensations will only be derived from the point cut. Therefore I regard the rubber dam, as capable of performing a double office. I am sure it is quite unnecessary for me to call the attention of those of you who are about to graduate from this institution, to the *absolute* necessity of using the rubber dam, in the large majority of your operations; and the only excuse I offer for so doing, is that I know very reputable men who are to-day practicing without the aid of the rubber, simply because they always have, and probably always will. Neither they nor their patients know the loss they are sustaining. In this surely, ignorance is not bliss.

Treat your patients as though you expected them to appreciate your ability, and your care for their welfare. Some will give unmistakable evidence of their non-appreciation; but let this make no difference in the conscientious performance of your duty toward them.

When one takes your chair for a general diagnosis of the oral cavity, make it thorough, and I have found a very convenient help in the use of diagrams of the teeth, that I keep for the purpose, which correspond with one in my Ledger. This I lay before me, and beginning with the superior centrals, inspect carefully with my explorers, first one side of the arch, and then the other, and wherever an imperfection presents itself, with my pencil I make a dot upon the tooth on the diagram, corresponding with the one in the month. By this means I am able to give the inquirer a very correct idea of the condition of the teeth, as well as preserve for my own benefit the chart, which will enable me to begin my operating understandingly, when the time arrives, without spending time for a second examination.

Do not attempt the most tedious and painful operations first, especially if your patient is given to timidity and nervousness. As a rule, your better plan will be to spend the time of the first sitting in thoroughly cleaning the teeth, which will enable you to detect more accurately the existing defects. Converse with your patients mean while, instructing them as to what they may expect, thereby preparing them to meet the operations that are to follow, without so much of fear and painful disappointment as is often manifest. This applies more particularly to those whose teeth have been neglected in youth, and they have reached mature years without much dental knowledge. To those who have regularly consulted a dentist from childhood, this will be less important.

Do not neglect to give your patients, who are parents, oft repeated reminders of the necessity of sending in their children, early and often; for in this is the key note to the successful treatment of the teeth of the human race. But do not rest your case here, whenever an opportunity presents, ever be ready to give your patients, particularly parents, good wholesome advice as to diet, cleanliness, exercise, and matters generally pertaining to health.

When you are requested to make an estimate upon an operation, or series of operations, in advance of their execution, refrain from placing a lower value upon them, than you think they should command, for the purpose of securing patronage; for unless you are endowed with a keener sense of exact justice than most men, you will be in danger of doing violence to your reputation.

Do not allow a brother practitioner to establish or regulate your fee bill. I am in favor of dropping from the dental vocabulary the word fee-bill entirely; it is often the cause of misleading the public, and doing injustice to yourself. Exact, as a remuneration for your services, what you regard as your just due, and you are the best and only judge of that.

While I do not countenance what are called low prices, I have no sympathy with extortion. Do not endeavor to justify the charges made, by representing to your patients an extravagant cost of material used; place the estimate upon your *time*, and not your material.

Beware of giving patients license to drop in at any time, and if you are not busy you will serve them. As a rule, operate by appointment only; except to relieve the suffering.

In making an appointment, give your patient as nearly as possible, the length of time you will require their presence, both for their benefit and your own; and you will find it will require some skill, so to arrange your appointments as to prevent the time of one patient overlapping that of another.

Be always careful not to disappoint your patients. Promptness in meeting your engagements, will greatly influence them to the same: which will be the means of saving you much annoyance and valuable time. Also never promise a piece of work till such a time as you are reasonably sure of having it done, better give yourself a day more than you think you will need than to promise what you cannot fulfil. Very few people will bear patiently being put off with this or that flimsy excuse. Teach your patients that they can rely on your word in small things as well as great.

To those of you who are about to receive your certificates of qualification, I would say, do not betray the weakness of supposing that because you have received your diplomas, you have reached the acme of dental knowledge; for as we, who have been in the profession many years, take a retrospective view, and behold the rapid advances that have been made, the interesting problems that have Leen solved, and imagine what *may* and *must* be stored up in future for those who will seek for it, we can hardly lay claim to more than the border land of scientific truth. Therefore, I say, do not lay aside your *studies*, like the conqueror, who lays aside his sword to rust because the victory is won, for in *this* the enimence has never been attained and probably never will be this side of the Millennium.

I think we can well appropriate to ourselves the words of Paul to the Corinthians, when he says, "Know ye not that they who run in a race, run all, but *one* receiveth the prize. So run that ye may obtain, and every man that striveth for the mastery is temperate in all things."

If you have entered the profession in good faith, you will find the practice of its replete with pleasantries, good associates, the relief of the suffering, the happy expression on the faces of very many for whom it will be your privilege to restore the broken, and replace the lost monuments they have so much prized in former days.

You must be prepared however to receive praise unmerited and frowns undeserved. Do not be too much affected by what may be said of you; remember also that the words *reputation* and *character* convey very different meanings. A man may bear a good reputation and have a bad character, but a man with a good character, cannot well bear a bad reputation.

In the practice of your profession, resolve upon honesty of purpose in all that you do, *not* because honesty is the best policy, for if *policy* is the only incentive to honesty, a man may have the reputation of it, and be a knave at heart.

Be honest to your patients and to society, for you are sure to be judged by an ever watchful public, by the company you keep.

Be ready to express your political preferences when occasion demands *only*. Avoid entering into politics, further than duty calls.

While you are engaged in the practice of dentistry, do not give any portion of your time to outside business occupations, for just in proportion as you withdraw your forces from your profession, will you deprive your patients of their just due.

Keep as well posted in everything pertaining to the advancement of the profession, as your time and means will permit. Supply your library with all the popular journals and new works on topics of interest; and not only *have* the works but *study* them; and strive by comparing the theory and practice off different men, to get new ideas which you can apply in your own case. By adopting this course, you will more and more become convinced that you are never too old to learn from others.

Associate with brother practitioners to this end; and avail yourselves of every honorable means to gain new information, either in dentistry or medicine, for the more you know of the latter in connection with *your* branch, the nearer will you approach to the knowledge you desire to attain. You will doubtless be called upon frequently to diagnose cases in which you would be almost in the dark, were you ignorant of the physiological bearings in them. And now, in the beginning of your practice, while your time is comparativly unoccupied, spend all your available time in pursuing those collateral branches, which have a direct bearing on your specialty.

I hope it is entirely unnecessary for me to warn you against the mean and petty jealousy, which frequently exists between professional men; and which is often the cause of preventing the intercourse, and interchange of ideas, that are so vital to growth. How *often* is it the case, that professional men are found, using the same old instruments they commenced practice with; and with no idea of the value of the appliances that have from time to time come into use. On inquiry you will find they never attend the dental associations, and rarely go into a neighboring office: but have been content to plod along in the same rut for years, with no desire for a "more excellent way." Cultivate broad, and liberal views, and be not over anxious to throw aside a new idea *because* it is *new* or startling, but give to every one's views, the consideration and respect you would desire for your own: and only cast them away, when, after mature thought, you decide they are impracticable; you *may* be entertaining an angels unawares.

And now your career is before you. It is as yet only in embryo, and in a plastic condition, so that by your strong, good sense, and adherence to the promptings of your better nature, you can mold it to your will.

Do not let the "slings and arrows of outrageous fortune," daunt you, but in the possession of an inflexible determination to *do right*, and "hold fast that which is good," have courage to "take arms against a *sea* of troubles, and, by *opposing*, end them."

Never make a retrograde movement, so that at the end of a long and useful life, you may take a retrospective view, with a happy consciousness of having aided in the progress of science, and thus in benefitting the human race; and if this is your record, you cannot have lived in vain. And last, but most important of all, strive to live *such lives*, as shall be accepted of Him to whom you are indebted, for all the powers you possess.

